# Rice farmers’ perceptions and acceptability in the use of a combination of biolarvicide *(Bacillus thuringiensis* var. *israeliensis)* and fertilizers application for malaria control and increase rice productivity in a rural district of central Tanzania

**DOI:** 10.1186/s12936-019-2697-y

**Published:** 2019-03-12

**Authors:** Humphrey D. Mazigo, Isolide S. Massawe, Susan F. Rumisha, Eliningaya J. Kweka, Leonard E. G. Mboera

**Affiliations:** 1 Department of Medical Parasitology, School of Medicine, Catholic University of Health and Allied Sciences, P.O. Box 1462, Mwanza, Tanzania; 2 National Institute for Medical Research, Tanga Research Center, Tanga, Tanzania; 3 National Institute for Medical Research Headquarters, Dar es Salaam, Tanzania; 4 Tropical Pesticides Research Institute, P.O. Box 3024, Arusha, Tanzania; 5 Southern African Centre for Infectious Diseases Surveillance, Chuo Kikuu cha Kilimo, Sokoine, Morogoro Tanzania

**Keywords:** Biolarvicide, Fertilizer, Malaria, Larviciding, Rice harvest, Community acceptability, Willingness to pay, Tanzania

## Abstract

**Background:**

The use of larval source management as a supplementary intervention for malaria control has not been widely used in rural Africa due to perceived high costs and complex logistics. To reduce the cost of larviciding in rice farming communities, concurrent application of biolarvicides and fertilizer in rice fields was introduced to control malaria vectors larvae and improve rice grain yields. The present study determined rice farmers’ perceptions and acceptability in the use of a combination of biolarvicide and fertilizers in farming practices.

**Methods:**

This was a qualitative study conducted among rice farmers at Kilangali village, south-central Tanzania. Semi-structured interviews and three focus group discussions (FGDs) were conducted with men and women who participated in the biolarvicide and fertilizer application project. The interviews and discussion focused on knowledge, attitudes and perceptions of participants on the use of the innovation in their farming practices and their willingness to pay for the innovation.

**Results:**

A total of 40 (mean age = 38.8 ± 10.12 years) rice farmers were involved in the study. Overall, all farmers agreed that it was possible to apply the two products concurrently with minimal challenges. The trust on the safety of biolarvicides on both human and paddy health was high. Respondents reported no challenge in preparation and applying the product in their rice fields. Over half (56.6%) of the participants reported an average decrease in mosquito density in their households and a quarter (26.6%) of them reported a decrease in mosquito population in their farms. Similarly, 93.3% of the participants reported that the intervention had reduced malaria risk in their households. In general, all participants expressed willingness to contribute to a biolarvicide and fertilizer programme and to use the approach in their farming practices.

**Conclusion:**

Community-based concurrent application of biolarvicides and fertilizer in rice fields was feasible and led to a perceived reduction in mosquito density. Willingness to pay for the larviciding/fertilizer approach was expressed by participants and they accepted to use the approach in their future farming practices. However, the impact of this approach on malaria transmission and rice grain harvest need to be evaluated in a large-scale programme.

## Background

Malaria remains as a single most common vector-borne diseases in the subtropical and tropical areas of the world [1]. Despite the fact that the global burdens of malaria morbidity and mortality have declined by 18%–48% in recent years [1], the sub-Saharan African (SSA) region still reports high number of malaria cases and deaths especially among children [1]. In Tanzania, malaria remain as number one mosquito-borne disease accounting for over one-third of hospital attendance and mortality [1–4]. The disease is estimated to cause over 16 million of clinical cases presenting at outpatient departments and over 100,000 deaths annually [1, 4]. Malaria transmission in Tanzania is not homogenous, with over 90% of the population living in areas where malaria is endemic. The rural population is disproportionally affected by the disease in comparison to other communities [2, 5, 6]. Malaria transmission varies with agro-ecosystem and rice irrigation farming communities are reported to carry the highest burden [5–10].

Malaria control in Tanzania mainly focus on strategies that targets adult mosquito vectors, which include the use of long-lasting insecticide-treated nets (LLINs) and indoor residual spraying [4, 6]. For malaria parasites control, the strategies focus on prompt diagnosis and early effective treatment using artemisinin-based combination therapy and intermittent preventive treatment using sulfadoxine–pyrimethamine (SP) during pregnancy [6]. However, the methods are challenged by growing malaria vector resistance to insecticide [11–13] and anti-malarial drug resistance [14–16]. In that light, supplementary malaria vectors control interventions targeting mosquitoes at their early stages such as larval source management is highly needed as a component of an Integrated Vector Management (IVM) strategy [17].

Despite the historical success of malaria vector control through larval source management [18–21], the approach has received insignificant attention and it plays a very minor role in malaria control agendas in SSA [22]. Mosquito larviciding using highly specific toxins producing bacteria, *Bacillus thuringiensis* var. *israeliensis* (Bti) and *Bacillus sphaericus* (Bs) is a promising supplementary measure [17]. The advantage of using larvicides is that it targets immobile stages of the vector in large density in easily accessible breeding sites such as paddy fields [23–25]. In addition, larval stages cannot change their behaviours like adult vectors [26, 27], and to date, there are no reports on development of resistance to the currently used biolarvicide. The effectiveness of Bti and Bs in reducing malaria larvae and adult density with subsequent reduction in malaria cases has been documented in many regions of SSA [20, 21, 23, 28, 29].

In term of cost, malaria vector larval source management is deemed as cost effective and competitive approach in relation to other alternative malaria control measures [29, 30]. Recent estimate in Burkina Faso reported that an average annual per capital cost of exhaustive larviciding with Bti during the high malaria transmission seasons were US$1.05 and spraying of 50% of most productive larval sources the cost were US$ 0.77 [30]. These cost were lower than US$3.80 and US$3.00 for anti-malarial drugs and LLINs respectively in SSA [30]. In a more recent study in central Tanzania [31], an average annual economic cost of biolarvicide intervention was calculated to be US$ 1.44 per person, per protected year. Based on the fact that larval source management cannot stand alone, the given costs allow this approach to be integrated into existing malaria control strategies. Available evidence has shown that, in areas where larval source management was used led to a significant improvement in malaria control [32, 33].

Recently, the World Health Organization (WHO) has recommended the use of Bti based larval source management in urban areas [32]. This excludes rural areas of which malaria is still a serious problem, and larval habitats are numerous and widespread [4]. The application of such intervention in these settings will require time, man-power and probably high cost. To overcome some of these challenges especially reaching some of the potential malaria vectors breeding sites created by human economic activities in rice farming agro-ecological system, an approach which actively involve local community to apply biolarvicides is highly recommended [19, 33–36]. In that context, this study adopted rice farmer’s fertilizer application skills to deploy biolarvicide (Bti) in rice fields in a rural district of central Tanzania. The objective of this study was to determine farmers’ perceptions and acceptability in the use of a combination of biolarvicide and fertilizers application for malaria control and increase rice productivity in a rural district of Kilosa in central Tanzania.

## Methods

### Study area and selection of study participants

**Fig. 1:**
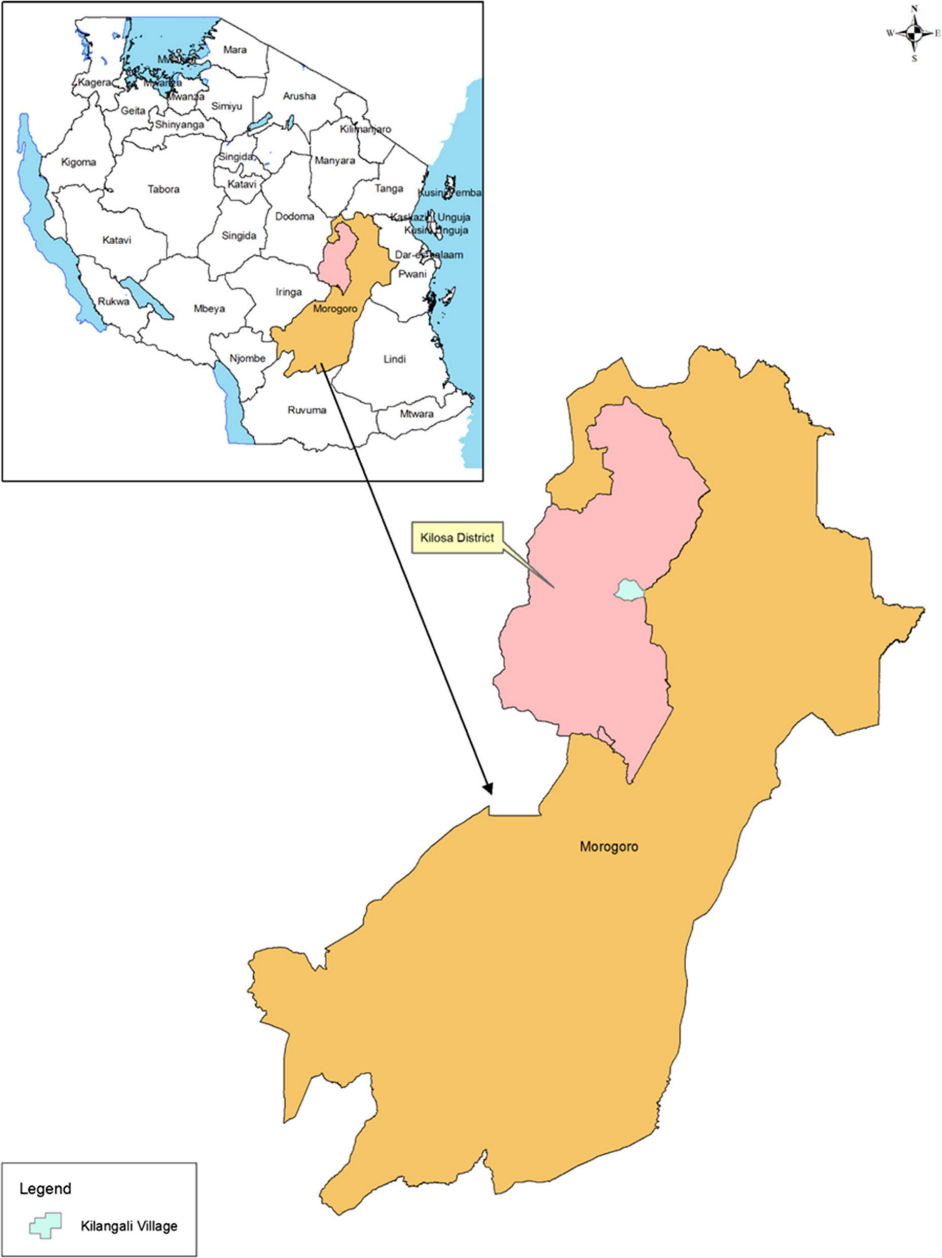
A map of Tanzania showing the location of Kilosa district

This study was carried out at Upogoroni sub-village, Kilangali village (6°58′0″ S; 37°5′0″E), Kilosa District of central Tanzania (5°55′–7°53′ S; 36°30′–37°30′ E) (Fig. 1) in July 2016. The village is located in the south-eastern part of the district and is characterized by swampy flatland and wetlands lying on the Kilangali alluvial basin. The village is bordered by a large Kilangali Rice Seed Farm Irrigation scheme totalling 1200 hectares. Within the Kilangali village there are six sub-villages, which include Mlegeni, Kisiwani, Makuluwili, Kwamtunga, Upogoroni and Chamwino. The village population was estimated at 3500 inhabitants.

**Fig. 2:**
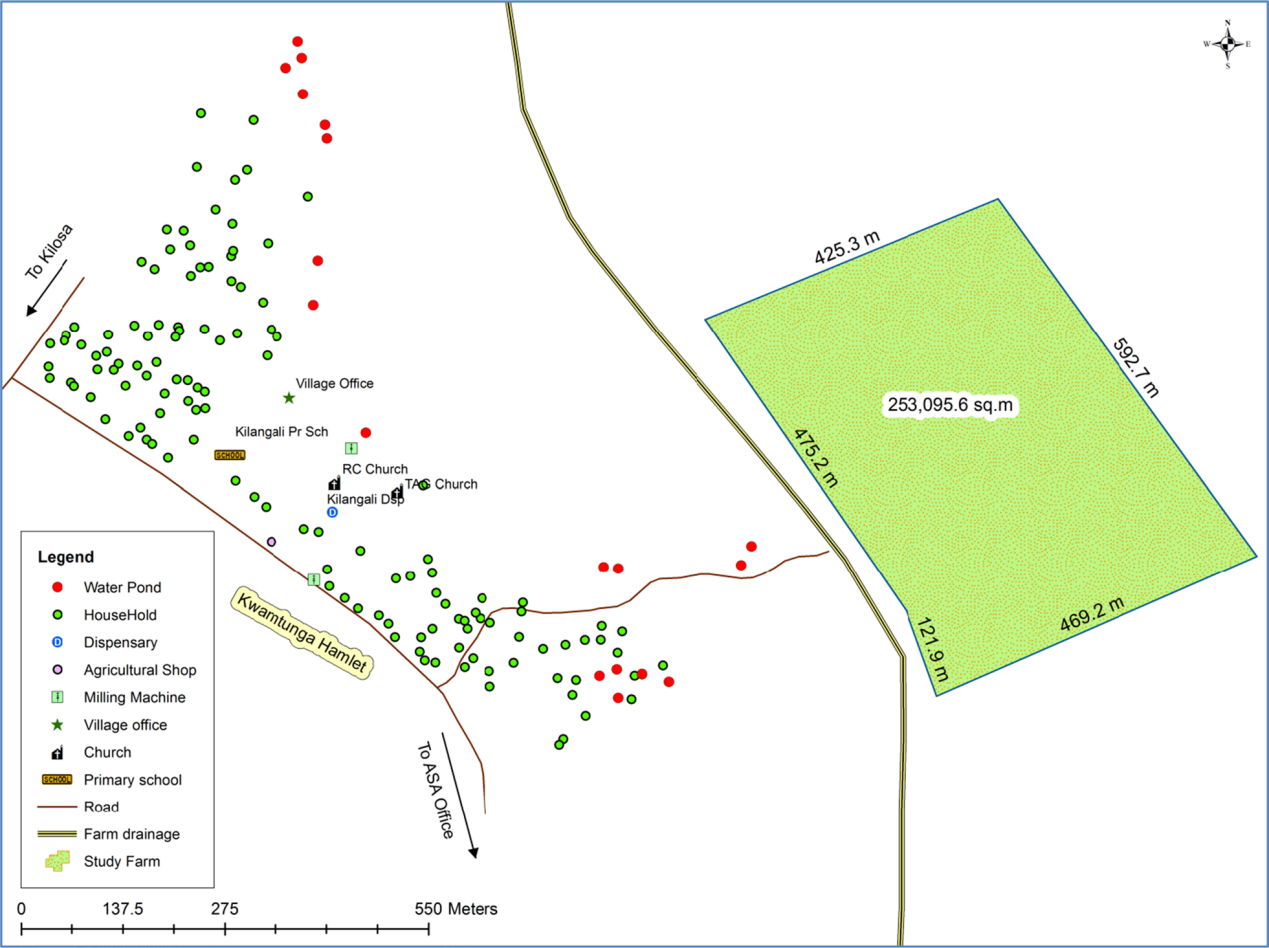
A map of Kilangali village at Kilosa district showing the location of the farm block where biolarvicides application was done to control malaria transmission and increase ride grain output

A block of 253,095.6 m^2^ [37] located close to Upogoroni sub-village (Fig. 2) was purposively selected and used for the biolarvicide and fertilizer application intervention (Fig. 2). All farmers owning a plot in the selected area were eligible for participation and included in the study. Inclusion criteria were owning a plot in the selected block and having a farm plot ready for paddy planting by February 2016. Of these, 31 farmers used a mixture of biolarvicide and fertilizer during the farming season of February–May 2016. Nine (9) farmers used neither a mixture of biolarvicides and fertilizers nor fertilizer (a common farming practice in the area). On average, each of these farmers owned one-acre (0.4 hectare) plot.

### Preparation and application of biolarvicide and fertilizer mixture

**Fig. 3:**
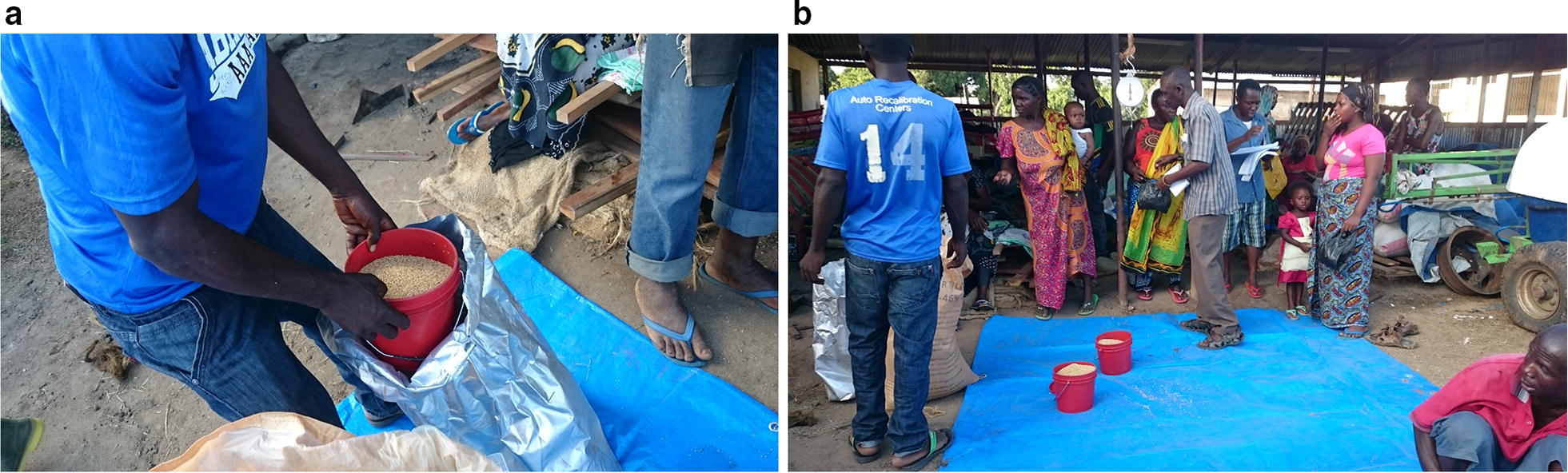
**a** Biolarvicide-BTI and **b** farmers are meeting to collect biolarvicide and fertilizer at Kilangali Rice Seed farm, Kilosa district, central Tanzania

**Fig. 4:**
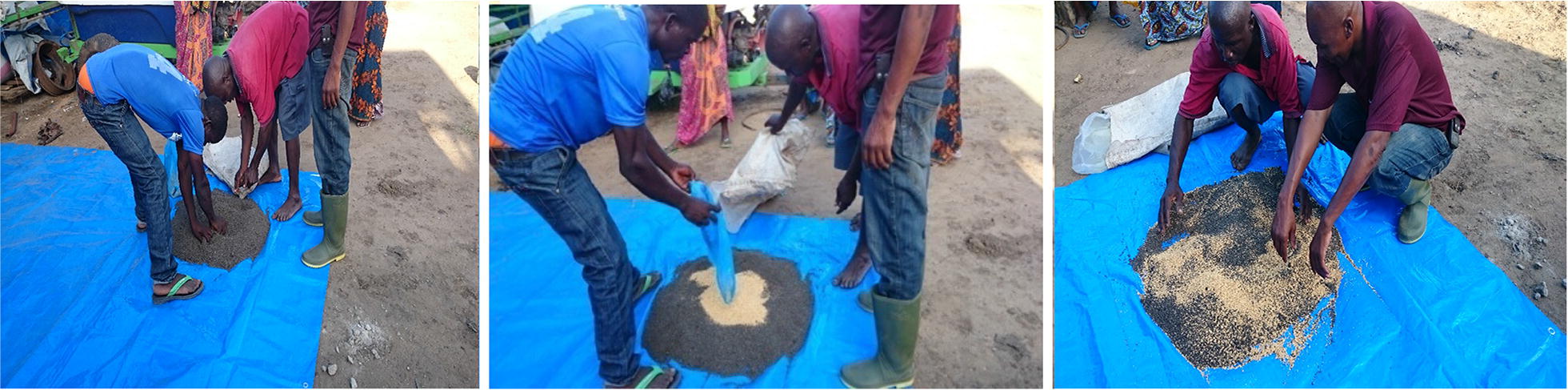
Rice farmers mixing biolarvicide-*Baccilus thuringiensis var israeliensis* and DAP fertilizer

Commercially available biolarvicide, in form of corn granules (*Bacillus thuringiensis* subspecies *israeliensis*-Bti, strain AM-65-52) (VectoBac, Lot no. 251-997-N8, Valent Biosciences Corporation, USA) was used. Di-ammonium phosphate (DAP) and urea fertilizers, were used as basal and top-dressing fertilizers, respectively, by farmers. Prior to the application of biolarvicide and fertilizer mixture, the rice farmers were trained on how to mix and apply biolarvicide and fertilizer (Fig. 3). To prepare biolarvicide and fertilizer mixture for one hectare, a 3.7 kg of Bti was mixed with 49.4 kg of DAP fertilizer for basal dressing and the same amount for urea fertilizer for top dressing. The application skills and timing of Bti and fertilizer was based on the results of our semi-field experiment which showed that at day 7–10 were the best time for applying Bti and after these days, the paddy fields were re-populated by mosquito larvae. The mixture of Bti and fertilizer was prepared by the farmers themselves under the supervision of the investigators (Fig. 4). The main application timing were (i) At the tilling stage (1 day after transplanting; (ii) At the panicle initiation stage (28 days before heading), and (iii) At the booting stage (60 days before heading) [38]. On day zero, farmers applied a mixture of biolarvicide and DAP fertilizer for basal dressing. On day 28 and day 60, a mixture of biolarvicide and urea fertilizer was applied for top dressing. Then, after every 7 days, farmers re-applied biolarvicides in their paddy farms to control mosquito larvae abundance. A sowing method was used to apply a mixture of biolarvicide and fertilizer. Each rice farmer applied the mixture by hand from a bucket as she/he walked from the edges of the plot.

### Data collection

#### Quantitative study

A pre-tested structured questionnaire was used during individual interview with male and female rice farmers. The interview was held at the village office located near the Upogoroni sub-village, in rooms that were accessible and convenient for the study participants. The following information were collected during the interview;—knowledge and awareness about the biolarvicides, attitudes and perceptions towards safety and effectiveness of biolarvicides, experience regarding the use and acceptability of biolarvicides and fertilizer combination as well as willingness to include payments for the biolarvicides and fertilizer combination on routine farming plan to enhance malaria control and increase rice grain productivity. The average time for the interview per one study participants was 20 min and the interviews were conducted in Kiswahili language.

### Qualitative study

#### Focus group discussions

After the quantitative survey was completed, Focus Group Discussion (FGD) was conducted using almost similar topics to what was presented in the interview section above. The following aide memoirs were asked during the discussion: what were the farmer’s experiences on preparation of biolarvicide and fertilizer mixture and application, what were the challenges faced during this process; what were their impression and perception on the exercise and what were the perspectives of other community members and their willingness to use the innovation as farming practice in the future. The narratives during the FGD were recorded using a digital audio recorder and the recordings were complemented by short hand notes. The discussion lasted from 45 to 60 min. All interviews were conducted in Kiswahili language by two social scientists.

#### Data processing and analysis

Audio recorded data were transcribed, translated from Kiswahili to English and double checked for clarity prior to analysis. The data were analysed manually using thematic framework analysis which involved ordering, structuring and interpreting the collected data. The themes identified included experienced challenges on use of the intervention, perceived impact of the biolarvicides and fertilizer mixture on malaria disease and mosquito density in households and farming area, trust on safety of the biolarvicides and fertilizer mixture and productivity, benefit of the innovation, willingness to use the innovation in their farming practices in the future and willingness to pay for the biolarvicides and fertilizer mixture. From the structured questionnaire data, descriptive summary statistics were generated using Stata package version 15 (StataCorp, 4905 LP, Lakeway Drive, College Station, Texas, USA). Statistical significance was considered at 5% level.

## Results

### Quantitative study

#### Demographic characteristics of study participants

A total of 40 (mean age = 38.8 ± 10.1 years) rice farmers were selected to participate in the present study based on the selected area for a period of 4 months (February–May, 2016). Of these, 31 (females = 56.7%; males = 43.3%) used a mixture of biolarvicide and fertilizer; their mean age was 40.8 ± 11.73 years. Nine farmers (female 55.6% and male 44.4%) did not use a mixture of biolarvicide/fertilizers. All farmers reported to have completed primary school education level (7 years of primary school).

#### Rice farmers’ knowledge, attitudes and perception

Among the 31 farmers who used biolarvicide and fertilizer mixture, 30 participated in the individual interview using a structured questionnaire. Almost all farmers (96.7%) agreed that their plots received enough rainfall during the farming season and they had satisfactory rice grain harvest. All participants reported to trust that biolarvicide used had no effects on human, paddy and animal health. No farmer reported noticeable changes (perceived side effect of biolarvicide) in the paddy plants or on their skin after applying the biolarvicide and fertilizer mixture. Other responses of rice farmers are shown in Table 1.

**Table 1:** Rice farmer’s responses to the quantitative questionnaire during the interview

S/no	Questions	Responses (N = 30)	
		Yes (n, %)	No (n, %)
1	Know about the biolarvicide and fertilizer application project	100 (30/30)	
2	Know about implementation of biolarvicide fertilizer project	100 (30/30)	
3	Participated in the implementation of biolarvicide and fertilizer project	100 (30/30)	
4	If yes at question 1–3, did you trust that biolarvicide have no effect on paddy plant health	100 (30/30)	
5	After using biolarvicide and fertilizer, did you notice any health effect on paddy health?		100 (30/30
6	Do you think use of biolarvicide and fertilizer have increased your income compare to the past season	100 (30/30)	
7	Did you experience any challenges in preparation and application of biolarvicide and fertilizer?		100 (30/30)
8	Do you think the use of biolarvicide and fertilizer have decreased mosquito density in your farm?	26.6 (8/30)	73.4 (22/30)
9	Do you think the use of biolarvicide and fertilizer have decreased mosquito density in your household?	93.3 (28/30)	6.7 (2/30)
10	Do you think use of biolarvicide and fertilizer have decreased the risk of malaria to your household and the community in general	93.3 (28/30)	6.7 (2/30)
11	Are you ready to contribute for biolarvide and fertilizer program in the future?	100 (30/30)	
12	Are your ready to use the biolarvicide and fertilizer mixture in future farming practices?	100 (30/30)	

#### Acceptability to use the innovation in their farming practices

All farmers agreed that, after participation in the study, their rice harvest per area had increased significantly compared to previous farming season (2015), which they did not apply fertilizer in their farms. They attributed the increase in their harvest to the use of biolarvicide and fertilizer mixture. About a quarter (26.6%) of the farmers, reported a decrease in mosquito density in their farming area compared to previous farming seasons which allowed them to work from early hours of morning to late evening hours (6 a.m.–6 p.m.). When asked if they experienced any challenges from preparation and applying the biolarvicide and fertilizer mixture, all of them agreed that they did not experience any challenge.

The vast majority of farmers (93.3%) responded that, the use of biolarvicide and fertilizer mixture had effects on mosquito density in their households. Majority (56.6%) estimated that there was an average decrease in mosquito density in their household compared to the previous farming season. Similarly, 93.3% of the farmers believed that the use of biolarvicide and fertilizer mixture had reduced a risk of contracting malaria among members of their household.

#### Willingness to pay for biolarvicide and fertilizer mixture

When asked if they were willing to contribute a specific amount of money after every 3 months for the biolarvicide and fertilizer application programme to enhance malaria control and increase rice grain productivity, all farmers (100%) agreed to contribute. Similarly, all farmers agreed to use biolarvicide and fertilizer mixture in the coming farming season to increase rice grain productivity and reduce malaria transmission if the use of this product will be registered and recommended for future use.

### Qualitative study

#### Challenge(s) on the use of biolarvicides and fertilizer mixture

The majority of the participants both in the quantitative and FGDs studies reported that they did not experience any challenge(s) in preparing and applying biolarvicide and fertilizer mixture in their paddy farms. The preparation and application process were described to be easy to perform and farmers acknowledged the involvement of the agricultural extension officers.

“…*it was not difficult to carry, mix and apply biolarvicide and fertilizer in my farm because we had instructors (Agricultural expert) who helped us to carry out the work…*’’ (P02, F).

“… *I mixed 40* *kg of fertilizer and 4* *kg biolarvicide and applied to my farm…”* (P05, M).

#### Perceived effects of the biolarvicides and fertilizer mixture on malaria cases

Numerous perceived positive effects and benefits of biolarvicides and fertilizer mixture were mentioned by participants, not only for the study participants but also for communities as a whole. Study participants mentioned that the use of the innovation resulted in the reduction of malaria cases and mosquito density in their households.

“…*this season in my household, malaria cases have decreased and I can say there was no malaria case at all*…” (P23, M).

*“.*..*mosquito density has decreased and malaria cases have also decreased significantly too…”* (P10, F).

“…*I would say this project is good as it helps to fight and kill mosquitoes and try to eradicate malaria from our area* …” (P04, F).

#### Perceived effects of the biolarvicides and fertilizer on mosquito density in paddy farms

The perceived benefits of biolarvicides and fertilizer extended beyond the reduction of malaria cases and mosquito density inside the participant’s houses. During the interviews and focus group discussions, participants acknowledged that the intervention had decreased mosquito density in their farms compared to the previous farming season. Before the implementation of the intervention, working in the paddy farms were associated with mosquito bites which interfered their working hours.

*“…last season before we were using these product (biolarvicide and fertilizer) we couldn’t stay on our farms for long hours especially during the morning and late evening hours because of excessive mosquito bites. However, during this season after having used these product (biolarvicides and fertilizer) we were able to stay on our farms until seven o’clock in the evening because mosquito density decreased significantly ….”* (P18, M).

#### Impact of the biolarvicides and fertilizer mixture on rice harvest

Similar to the quantitative survey, participants in the FGD agreed that, after participation in the study, their rice harvest per cultivated area for this farming season increased significantly compared to previous farming season. In previous farming seasons, farmers did not apply fertilizer in their rice farms. The use of fertilizer has increased the output from the paddy plants.

“… *Before this project, rice harvests were low, but this year we had good harvest. For example, this season I have harvested 20 bags of rice from one acre…*” (P14, M).

“…*the harvest has been good compared to last year and the project has increased my harvest and income as I was able to harvest 40 bags of rice from my one and half acre farm…”* (P12, F).

#### Perception towards safety of biolarvicide and fertilizer mixture on human and paddy plant health

Prior to participation in the study, rice farmers had fears and concern about the safety of the biolarvicides and fertilizer mixture. However, after participating in the intervention, there was a high level of trust on the safety of biolarvicides and fertilizer mixture in terms of paddy plant’s health and productivity. This was revealed in quantitative and FGD studies. Risks were mainly discussed in terms of fear of unknown side effects to their paddy plants.

*“…we were worried before using the biolarvicide but after using it nothing happened…”* (P09, F).

*“…we were worried that our plants and soil will be affected but after using the mixture, nothing had happened and results were so good to the extent that we even wanted to stay in our farms…”* (P14, M).

#### Willingness to pay for the intervention

Both in quantitative and qualitative studies, participants demonstrated willingness to pay for the innovation. The perceived impact of the innovation on malaria cases, mosquito density and rice output were among the key driving factors on participant’s willingness to pay for the innovation.

Nearly all participants agreed to support the programme because they had already seen its positive impact.

“…*I’m ready to contribute because the intervention increased our rice production, reduced malaria transmission and allowed us to remain healthy with no malaria…”* (P22, M).

“…*we are ready to pay if the exercise will start on time. The product should be delivered on time especially around December when the farming season starts and if the exercise will continue until the dry season…”* (P15).

#### Recommendation to the project

Participants highlighted the need to scale-up the intervention in term of area covered and the duration of the project (the project was evaluated for only one farming season and covered a small area of Upogoroni sub-village). The advantage of scaling-up the intervention was based on improving paddy productivity and eradicating malaria in the village.

*“… the Project should cover large areas of the farms so as to give us a big impact on production …”* (P 21, M).

*“…the project should continue so that malaria can be eradicated from our families as well as in our village…”* (P17, F).

## Discussion

The findings of the current study revealed that, all participants accepted that biolarvicides when used alone or when mixed with fertilizer was safe to humans and paddy plants health. However, before they were engaged in the study, participants expressed their fears on the safety of the biolarvicides to their health and paddy plants. This was mainly related to the fact that majority of the farmers were unaware of larviciding as a means for controlling malaria and the effect on their paddy plants. Similar observations have been reported in a neighbouring district of Mvomero, Tanzania before the implementation of community larviciding activities to control malaria transmission [39]. In Rwanda, a number of concerns with regard to the safety biolarvicide (Bti) for human and paddy plants were raised by rice farming communities before implementation of the Bti application project using farmers [19]. Rice farmers feared that Bti would interact with fertilizer and other chemicals to kill *Rhizobium* bacteria which help to fix fertilizers in their farms [19]. However, after participation in the Bti implementation process, rice farmers in Rwanda acknowledged that Bti had no effects on human and paddy plant health [19]. The safety studies have shown that microbial larvicides are highly safe to human and animal health and highly specific to targeted organisms [23, 24, 40]. Together, these observations emphasize the need for community sensitization and giving clear information regarding the safety of interventions to human, animals, environment, and its effectiveness against the targeted organisms. This is important for community acceptability of the intervention and should be communicated during the community engagement [39].

In this study, the effect of the intervention on mosquito density and malaria risk was also explored. There was a general agreement and perception among participants about a reduction in mosquito density in their households and number of malaria cases had decreased compared to the last farming season. Interestingly, mosquito density was also reported to decline in their farms which allowed them to work from early morning to late evening hours compared to the previous seasons. Similar findings have been reported in rural Rwanda among rice farming communities [19]. Rice farmers in intervention areas mentioned reduction in mosquito abundances, mosquito bites in their farms and homesteads [19]. This allowed farmers to work without any interferences from mosquitoes [19]. Prior to Bti implementation, rice farmers in these communities mentioned that working in marshlands were associated with mosquito bites [19]. Microbial larvicides such as Bti mainly targets and kills malaria vectors and other mosquito’s species larvae in their breeding sites [21, 23, 24]. This results in decreasing adult malaria vectors and other mosquitoes densities in the targeted households [41]. A successful reduction in malaria vectors larvae density in the flooding plains of the Gambia led to a reduction in adult malaria vectors in households [41]. Similarly, in Dar es Salaam, Tanzania, larviciding using Bti successfully suppressed the household densities of the adult malaria vectors, namely *Anopheles funestus* and *Anopheles coustani* [33].

Overall, the findings of this study demonstrated that there was high acceptability of the interventions as well as willingness to pay for the programme. The main reasons for willingness to contribute were the effects of the intervention on health through perceived reduction in malaria transmission and its impact on paddy productivity. Similar findings were reported among rice farming communities in Mvomero, Tanzania who were willing to pay for a larviciding programme [39]. However, in Mvomero, households demonstrated willingness to pay across wide range of the given hypothetical price of contribution but the willingness decreased with an increased amount to contribute [39]. Authors also observed a significant association between willingness to pay for larviciding and trust on the safety of biolarvicides, education and wealth of participants [39]. In Sudan, socio-economic status was the most important determinant for communities’ willingness to pay for malaria intervention measures [42]. A high willingness to pay was demonstrated for insecticide treated mosquito nets and insecticides residual sprays, with larviciding being the least [42]. In Rwanda, rice farmers agreed to finance the Bti programme through their respective cooperatives to ensure sustainability of the programme and through their labour forces [19]. The willingness of malaria endemic communities to pay or to contribute to intervention measures present the concept of local financing of intervention programmes against malaria which if well designed will ensure sustainability of malaria control programmes at local levels [42].

On the other hand, study participants noted an increase in their rice harvest after participating in the study compared to the past farming season, in which they did not use fertilizer. The increase in rice grain was mainly related to the use of fertilizer and not because of including biolarvicide into fertilizers. As expected, the use of fertilizer in farming activities tends to increase plants productivity per given area [43]. This approach of concurrently applying biolarvicide and fertilizer in paddy fields has double impact in controlling malaria transmission and increase rice productivity. It ensures food security and reduce malaria incidence among rice farming communities. Thus, it was not surprisingly to note the high rate of farmer’s acceptability to use the approach and willingness to participate in the future. Farmers also commented on the need to increase the coverage areas to increase rice productivity. This also will have impact on malaria transmission through covering a wide range of human-made malaria breeding sites [29].

A number of recommendations were given out by participants to improve the outcome of the intervention. Farmers commented on the need to increase the coverage areas so as to achieve maximum effects of larviciding on malaria vectors densities, malaria transmission and rice output [25, 33, 44]. The large-scale coverage of all potential malaria vectors breeding sites will results into reduction of malaria cases. This study covered only an area of 253,095 m^2^. The extension of the biolarvicides application programme to dry season were also raised by study participants. Similar concerns and recommendation were raised by rice farmers in rural Rwanda [19]. Large scale application of Bti in potential malaria vectors breeding sites in Western Kenya [45] have demonstrated a reduction in larvae and adult malaria vectors densities by > 90% [29]. Similarly, larviciding during the dry season in Dar es Salaam resulted into significantly lowering the prevalence of malaria, larvae and adult vectors density [20].

In general, community participation is an important tool for successful designing, implementation and evaluation of the impact of malaria interventions with the aim of increasing ownership and sustainability of the control programmes [19, 35]. Involvement of the communities living in malaria endemic areas as a tool to deliver interventions has provided a renewed interest and showed that through this approach even communities living in remote areas can be reached and covered by interventions [35]. Thus, community mobilization and their active participation to implement the intervention are key elements for sustainability of the intervention programme [35]. On the other hand, the results of the present study and those of other authors [19] highlight the importance of multi-sectoral engagement between the civil and water engineering, agricultural and health sectors towards implementation of different malaria intervention measures [46].

## Conclusion

The finding of the present study shows that integrating biolarvicide into fertilizer application skill among rice farmers is possible and acceptable by rice farming communities. The use of biolarvicide and fertilizer mixture is potential complementary malaria intervention in rice farming communities. However, the impact of this approach on both malaria transmission and rice grain output remains to be studied at large scales before it can be widely accepted into malaria control programmes.

## Abbreviations

WHO: World Health Organization
SSA: sub-Saharan Africa
LLINs: long-lasting insecticide-treated nets
ITNs: insecticide-treated nets
SP: sulfadoxine–pyrimethamine
IVM: Integrated Vector Management
Bti: *Bacillus thuringiensis* var *israeliensis*
Bs: *Bacillus sphaericus*
NMCP: National Malaria Control Programme
DAP: diammonium sulphate
FGD: Focus Group Discussion
F: female
M: male
P: participant
